# Prognostic Value of Standard Heart Failure Medication in Patients with Cardiac Transthyretin Amyloidosis

**DOI:** 10.3390/jcm13082257

**Published:** 2024-04-12

**Authors:** Fabian aus dem Siepen, Selina Hein, Eva Hofmann, Christian Nagel, Stéphanie K. Schwarting, Ute Hegenbart, Stefan O. Schönland, Markus Weiler, Norbert Frey, Arnt V. Kristen

**Affiliations:** 1 Department of Cardiology, Angiology and Respiratory Medicine, University Hospital Heidelberg, Im Neuenheimer Feld 410, 69120 Heidelberg, Germany; selina.hein@med.uni-heidelberg.de (S.H.); eva.hofmann@med.uni-heidelberg.de (E.H.); christian.nagel2@med.uni-heidelberg.de (C.N.); stephanie.schwarting@med.uni-heidelberg.de (S.K.S.); norbert.frey@med.uni-heidelberg.de (N.F.); arnt.kristen@med.uni-heidelberg.de (A.V.K.); 2 Department of Hematology, University Hospital Heidelberg, 69120 Heidelberg, Germany; ute.hegenbart@med.uni-heidelberg.de (U.H.); stefan.schoenland@med.uni-heidelberg.de (S.O.S.); 3 Department of Neurology, University Hospital Heidelberg, 69120 Heidelberg, Germany; markus.weiler@med.uni-heidelberg.de

**Keywords:** amyloidosis, ATTR, transthyretin, heart failure therapy

## Abstract

**Introduction**: Cardiac transthyretin amyloidosis (ATTR) is a progressive, fatal disease leading to heart failure due to accumulation of amyloid fibrils in the interstitial space and may occur as a hereditary (ATTRv) or wild-type (ATTRwt) form. Guidelines recommend the use of ACE inhibitors (ACEis) and beta-blockers (BBs) as heart failure therapy (HFT) in all patients with symptomatic heart failure and reduced ejection fraction, independent of the underlying etiology. However, the prognostic benefit of ACEis and BBs in ATTR has not been elucidated in detail yet. We thus sought to retrospectively investigate the outcome of patients with ATTRwt or ATTRv under HFT. **Methods:** Medical records of 403 patients with cardiac ATTR (ATTRwt: n = 268, ATTRv: n = 135) were screened for long-term medication as well as clinical, laboratory, electrocardiographic and echocardiographic data. Patients were assessed between 2005 and 2020 at the University Hospital Heidelberg. Kaplan–Meier analysis was used to analyze potential differences in survival among different subgroups. **Results:** The mean follow-up was 28 months. In total, 43 patients (32%) with ATTRv and 140 patients (52%) with ATTRwt received HFT. Survival was significantly shorter in patients receiving HFT in ATTRv (46 vs. 83 months, *p* = 0.0007) vs. non-HFT. A significantly better survival was observed in patients with comorbidities (coronary artery disease, arterial hypertension) and HFT among ATTRwt patients (*p* = 0.004). No significant differences in survival were observed in the other subgroups. **Conclusions:** Survival analysis revealed a potential benefit of HFT in patients with ATTRwt and cardiac comorbidities such as coronary artery disease and/or arterial hypertension. In contrast, HFT should be used with caution in patients with ATTRv.

## 1. Introduction

Transthyretin (TTR) amyloidosis (ATTR) is a progressive, fatal disease caused by deposition of amyloid fibrils in different organs, leading to organ dysfunction and ultimately organ failure. Physiologically, TTR is a tetramer that transports thyroxine and retinol. When dissociated into monomers, the protein can form insoluble fibrils that accumulate as amyloid. Instability of TTR can either be caused by a mutation in the TTR gene or sporadically and is either referred to hereditary ATTR amyloidosis (ATTRv) or wild-type ATTR amyloidosis (ATTRwt) [1].

Approximately 140 different mutations in the *TTR* gene are potentially amyloidogenic with three different phenotypes: (a) predominant polyneuropathy (familial amyloid polyneuropathy, FAP), (b) predominant cardiomyopathy (familial amyloid cardiomyopathy, FAC) and mixed phenotype with involvement of the nerves and the heart. ATTRwt is characterized by sole cardiac manifestation and usually occurs in elderly men (>90% of the patients). Increasing awareness for ATTR cardiomyopathy (ATTR-CM) and improvement of the diagnostic possibilities led to a growing number of new diagnosed cases in the last few years. There are no specific symptoms for ATTR-CM; however, certain red flags can be suspicious and justify further diagnostic work-up. The medical history of ATTR patients often includes orthopedic conditions like bilateral carpal tunnel syndrome, spinal canal stenosis or distal biceps tendon rupture, in many cases years before the onset of heart failure symptoms; furthermore, ATTR-CM often coexists in patients with aortic stenosis [2] and is a common cause of heart failure with preserved ejection fraction (HF-pEF) [3]. When ATTR-CM is suspected, the diagnosis can be confirmed by endomyocardial biopsy or non-invasively by bone scintigraphy in combination with the exclusion of a gammopathy by immunofixation in serum and urine and normal free light chains in serum [4].

Both forms of the disease, ATTRwt and ATTRv with cardiac or mixed phenotype, lead to a restrictive cardiomyopathy with progressive, diastolic dysfunction. Median survival in ATTRwt without causative treatment has been reported to be from 2 to 6 years [5,6,7]. In ATTRv, survival depends on the underlying mutation and ranges from 3 to 12 years [5,8]. Several disease-modifying drugs are in development to stop or halt further amyloid deposition by stabilizing the TTR tetramer, inhibiting fibril formation or knocking down TTR synthesis. Since 2011, Tafamidis meglumine 20 mg, a TTR tetramer stabilizer, has been approved in Europe for the treatment of patients with FAP stage 1 [9]. In 2018, two RNAi drugs, Patisiran and Inotersen, were approved by the EMA and the FDA for the treatment of FAP in stage 1 and 2 [10,11]. Tafamidis 61 mg was approved as the first causative treatment for cardiac ATTRv and ATTRwt in 2020 to reduce mortality and cardiovascular-related hospitalization [12]. Moreover, there are Phase II and III studies evaluating the safety and efficacy of RNAi therapies and antibody therapies for treatment of ATTR-CM in progress.

Besides specific therapies for ATTR amyloidosis, several treatment options for patients with heart failure with reduced ejection fraction are available. The American and the European guidelines suggest the use of sacubitril or ACE inhibitors (ACEis) and beta-blockers (BBs) as standard heart failure therapy for symptomatic patients with reduced ejection fraction (HFrEF), independent of the etiology and combined with mineralo-receptor antagonists and SGLT2 inhibitors [13,14]. For patients with HF-pEF, no benefit for ACEis and BBs were observed, except for aldosterone antagonists [15] and SGLT2 inhibitors [16].

Cardiac amyloidosis usually leads to wall thickening with diastolic dysfunction and reduced longitudinal function, but often with normal radial function and global ejection fraction (EF), especially in early stages of the disease. In advanced stages, EF can be reduced; therefore, depending on the stage of the disease, cardiac amyloidosis can be considered as HFpEF or HFrEF. However, no patients with ATTR were included in the studies the guidelines for heart failure therapy are based on. The aim of this retrospective study was to elucidate the benefit of heart failure therapy in patients with ATTRwt and ATTRv with respect to the patients’ comorbidities.

## 2. Methods

### 2.1. Ethics

This retrospective study (S-091/2022) was approved by the local ethics committee. All patients gave written informed consent for the use of their acquired clinical data for research purposes.

### 2.2. Patient Cohort

To avoid influence of disease-modifying therapies, only patients prior to approval of Tafamidis in 2020 were included.

Other exclusion criteria were change in heart failure therapy during the observation period, participation in an interventional trial, treatment with sacubitril or gene silencers and predominantly neurological phenotype in ATTRv. Overall, 268 patients with ATTRwt and 135 patients with ATTRv were included in this retrospective analysis; 132 patients were excluded due to the above-mentioned exclusion criteria.

All patients were seen at the Heidelberg Amyloidosis Center between 2010 and 2020 and were diagnosed with ATTR and cardiac involvement at their first visit. Baseline characteristics are listed in Table 1. Follow-up was performed by phone assessment, either with primary care physicians or patients themselves. Only patients with full datasets and follow-up information were included.

### 2.3. Inclusion Criteria

- Age > 18 years;
- Confirmed diagnosis of ATTR-CM;
- Symptomatic heart failure with at least one hospitalization due to cardiac decompensation or symptoms of heart failure requiring treatment with loop diuretics.

### 2.4. Exclusion Criteria

- Participation in interventional trials;
- Treatment with disease-modifying therapies (silencers, stabilizers);
- Changes in heart failure medication during the observation period;
- Etiology for heart failure not primarily due to ATTR-CM, e.g., severe uncorrected valvular diseases, severe ischemic heart disease;
- Predominantly neurological phenotype in ATTRv.

### 2.5. Assessment of Organ Involvement and Confirmation of ATTR Diagnosis

The diagnosis of ATTR was made either by biopsy or alternatively by bone scintigraphy after the exclusion of monoclonal gammopathy (immunofixation and free light-chain assay in serum and urine) according to international standards. All patients were genetically tested for mutations in the *TTR* gene. Cardiac involvement was assessed by echocardiography and/or cardiac MRI and cardiac biomarkers. The presence of a typical ATTR polyneuropathy was confirmed by neurological examination including nerve conduction tests in all patients with ATTRv.

### 2.6. Clinical Examination

All patients underwent clinical examination including blood pressure and heart rate measurements, as well as 12-lead electrocardiography. Symptoms of heart failure were staged among the New York Heart Association (NYHA) from class 1 to class 4.

### 2.7. Laboratory Testing

Extensive blood analyses were performed including cardiac biomarkers. NT-proBNP was measured by Elecsys^®^proBNP, troponin T by Elecsys^®^2010 (Roche Diagnostics, Mannheim, Germany).

### 2.8. Echocardiography

Transthoracic echocardiograms were performed using commercially available ultrasound diagnostic systems (GE Healthcare, Milwaukee, WI, USA). All measurements were performed offline on a commercially available workstation (Centricity Cardiology CA1000 2.0, GE Medical Systems, Milwaukee, WI, USA).

### 2.9. Medication Survey

Medication plans of all patients were documented at the first visit and re-assessed in the follow-up visit. Heart failure therapies that were initiated before the visit to our center were not changed or interrupted during the observation period.

### 2.10. Statistics

Prism 8 (GraphPad Software V. 8.0.2 LLC) was used for statistical analysis, and a *p*-value of less than 0.05 was considered as statistically significant. Normal distributions were tested with the Kolmogorov–Smirnov test. We expressed categorical data as percentages and continuous variables as mean ± standard deviation. We used the *t*-test for comparison of two parametric variables and the chi-squared test for comparison of two categorical variables. Kaplan–Meier plots and the Log-Rank test were used for analysis of survival.

## 3. Results

### 3.1. Baseline Characteristics

In total, 135 patients with ATTRv and 268 patients with ATTRwt had complete datasets and were eligible for analysis. Naturally, ATTRwt patients were older compared to ATTRv patients (74 ± 6 vs. 63 ± 9, *p* < 0.0001) and more frequently of male gender (92% vs. 76%, *p* < 0.0001). Cardiac comorbidities were more frequent in patients with ATTRwt (arterial hypertension, AHT 62% vs. 32%, *p* < 0.0001; coronary artery disease, CAD 35% vs. 19%, *p* = 0.0002). Symptoms of heart failure, categorized according to NYHA classes, were also significantly different between ATTRv and ATTRwt patients (*p* = 0.001). Among ATTRv patients, 39% presented with cardiac phenotype and 61% with mixed phenotype; the most common mutations were p.Val50Met and p.Val40Ile. All baseline characteristics are given in Table 1.

### 3.2. Differences in Treatment

In total, 43 (32%) ATTRv patients were on heart failure therapy (HFT) consisting of BBs and ACEis. Patients on therapy had significant higher NT-proBNP levels (8598 ± 17,557 vs. 3562 ± 3808 ng/L, *p* = 0.02) and lower eGFR (69 ± 31 vs. 89 ± 33 mL/min × 1.73 m^2^, *p* = 0.002), whereas no significant differences in NYHA stages or echocardiographic parameters could be observed (Table 2). Among ATTRwt patients, 140 (52%) patients received HFT. No significant differences could be observed between patients with or without HFT (Table 2).

### 3.3. Subgroup Analysis

ATTRv and ATTRwt patients were divided into patients with and without HFT (Table 2). ATTRv patients revealed significant differences in NT-proBNP levels (8598 ± 17,557 vs. 3562 ± 3808 ng/L, *p* = 0.02) and eGFR (69 ± 31 vs. 89 ± 33 mL/min × 1.73 m^2^, *p* = 0.002) between both groups. No significant differences were observed in the ATTRwt cohort.

An analysis of the subgroups with cardiac and mixed phenotypes (Table 3) showed significant differences in systolic blood pressure (109 ± 21 vs. 121 ± 17 mmHg, *p* = 0.04) and NT-proBNP (7811 ± 8088 vs. 4208 ± 3972 ng/L, *p* = 0.046) for patients with cardiac phenotype and a significant difference in age (67 ± 6 vs. 62 ± 10 mL/min × 1.73 m^2^, *p* = 0.02) for patients with mixed phenotype.

### 3.4. Survival Analysis

The mean follow-up was 28 ± 27 months; median survival was 64 months in ATTRwt and 62 months in ATTRv.

**Figure 1:**
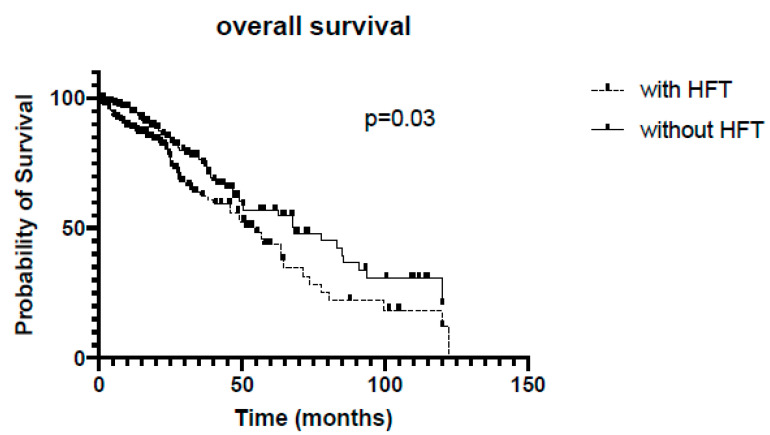
Kaplan–Meier analysis of overall survival. HFT = heart failure therapy.

**Figure 2:**
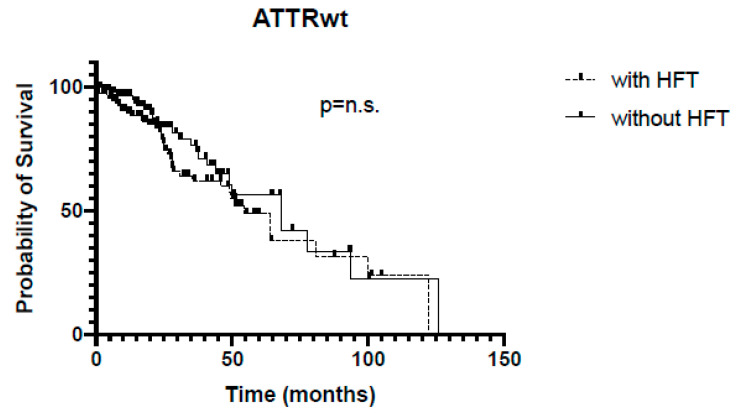
Kaplan–Meier analysis of survival in ATTRwt. HFT = heart failure therapy.

**Figure 3:**
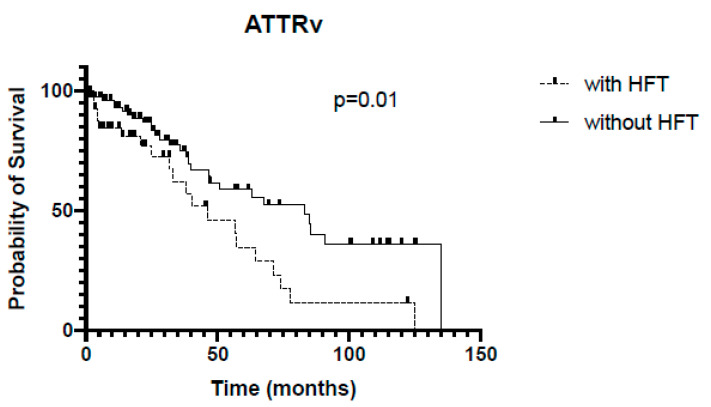
Kaplan–Meier analysis of survival in ATTRv. HFT = heart failure therapy.

In the whole cohort, patients without HFT survived significantly longer (68 vs. 54 months, *p* = 0.03, Figure 1). In the subgroup of ATTRwt, Kaplan–Meier analysis revealed no significant differences regarding survival between patients with or without HFT (54 vs. 67 months, *p* = 0.14, Figure 2), whereas among the ATTRv subgroup, survival was significantly shorter in patients receiving HFT as compared to patients not receiving HFT (46 vs. 83 months, *p* = 0.0007, Figure 3).

**Figure 4:**
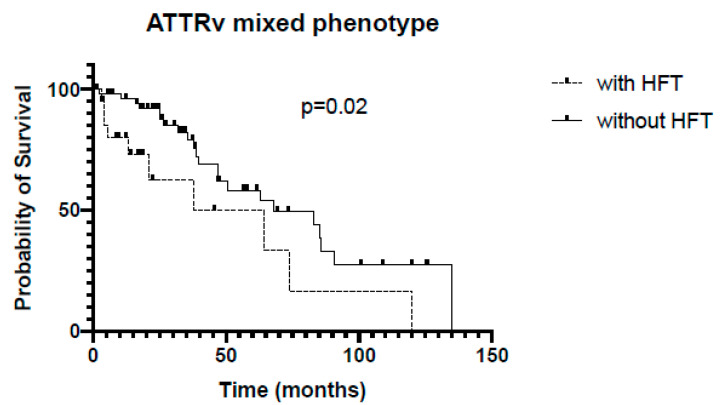
Kaplan–Meier analysis of survival in ATTRv subgroup, mixed phenotype. HFT = heart failure therapy.

**Figure 5:**
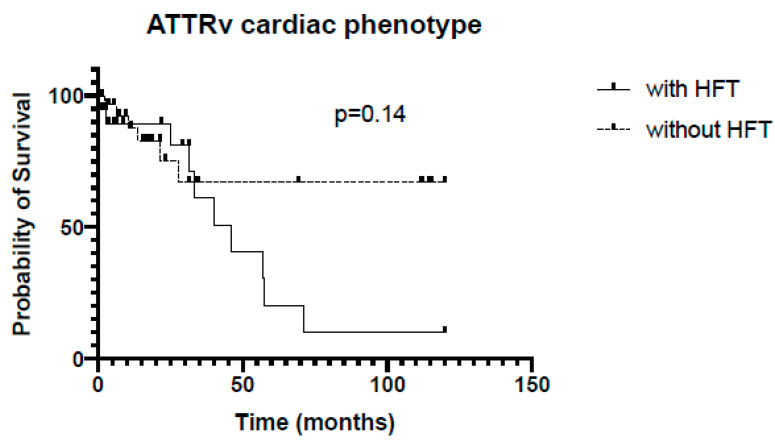
Kaplan–Meier analysis of survival in ATTRv subgroup, cardiac phenotype. HFT = heart failure therapy.

ATTRv patients with mixed phenotype and HFT had a significant shorter survival compared to patients without HFT (38 vs. 67 months, *p* = 0.02, Figure 4), but patients with cardiac phenotype with and without HFT showed no significant differences in survival (46 vs. 45 months, *p* = 0.14, Figure 5).

**Figure 6:**
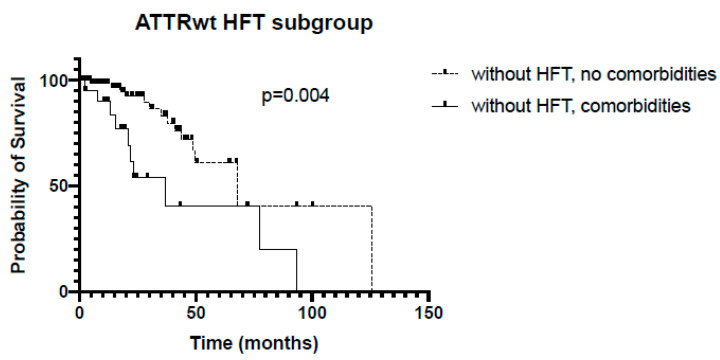
Kaplan–Meier analysis of survival in the subgroup ATTRwt with or without HFT. HFT = heart failure therapy.

ATTRwt patients with comorbidities (AHT and/or CAD) had significantly shorter survival if not receiving HFT (36 vs. 68 months, *p* = 0.004, Figure 6).

## 4. Discussion

This retrospective analysis revealed a potential benefit from standard HFT only for patients with ATTRwt and relevant cardiovascular comorbidities, e.g., CAD and AHT. Furthermore, HFT might have a negative impact on survival of patients with ATTRv and a mixed phenotype.

Therefore, overall, we could confirm the findings of a recent retrospective analysis by Ioannou et al. The investigators reported no relevant effect of treatment with ACEis and BBs in a large patient cohort of ATTR patients; interestingly, a positive effect for mineralocorticoid receptor antagonists (MRAs) could be observed, along with a positive effect for low-dose BBs in patients with a LVEF < 40% [17].

Up to now, the use of HFT has not been elucidated in prospective studies with ATTR patients. All large clinical trials that have been incorporated in heart failure guidelines did not contain any study on this topic in ATTR patients as these are not available yet. Moreover, almost all heart failure trials did not contain precise screening to exclude ATTR amyloidosis. Therefore, it cannot be excluded that these trials also contain patients with undiagnosed ATTR amyloidosis. In contrast, undiagnosed cardiac amyloidosis might also explain in part the negative results of previous studies on treatment of HFpEF.

The rationale behind the use of ACEis is the positive impact on myocardial remodeling [18]. Several studies demonstrated that the modulation of the renin angiotensin aldosterone system (RAAS) leads to a reduction in myocardial fibrosis and therefore improves outcome in patients with ischemic and non-ischemic heart failure with reduced ejection fraction (HFrEF). Thus, the combination of ACEis and BBs, as well as MRAs and Angiotensin Receptor–Neprilysin Inhibitors recently, became standard of care pharmacological therapy for HFrEF [13,14]. In contrast, no benefit was observed in patients with HFpEF. Only a few studies demonstrated a potential positive impact for the use of MRAs in HFpEF patients [15], leading to a class IIa recommendation in the AHA/ACC and ESC guidelines. Beyond that, no specific treatment is available for HFpEF, and guidelines focus on adequate therapy of comorbidities and exercise training. Moreover, the detailed pathophysiological background of HFpEF remains undetermined, resulting in a delay in the development of therapeutic strategies.

SGLT2 inhibitors recently changed the landscape of heart failure treatment [19]. Guidelines recommend the use of SGLT2 inhibitors for all types of heart failure in all stages from HF-rEF to HF-pEF [4,20]. So far, no prospective study investigated the efficacy of SGLT2 inhibitors for the treatment of ATTR-CM. But a first observational study demonstrated a good tolerability [21] and stabilization of NT-proBNP under therapy [22]. Especially due to their ability to improve fluid balance without compromising the systemic blood pressure, SGLT2 inhibitors should be considered as a therapy option for ATTR-CM patients; however, prospective studies are still missing for this specific disease.

Interestingly, recent studies suggested ATTR amyloidosis as a frequent cause of HFpEF. Gonzales-Lopez et al. demonstrated that 13% of a patient population with HFpEF suffered from ATTRwt [23]; in an autopsy study by Mohammed et al., ATTR amyloid was detected in 19% of the study population that was diagnosed with HFpEF antemortem [3]. The classification of ATTR cardiomyopathy is difficult due to its progressive nature with the continuous deterioration of cardiac function with progressive diastolic dysfunction and finally the impairment of systolic function. Depending on the duration of the disease, it can either be HFpEF or HFrEF. Therefore, ATTR cardiomyopathy does not fit clearly into one of the categories and remains an exceptional cause of heart failure. For that reason, no patients with ATTR were included in HFT studies intentionally, leading to non-existent data from randomized trials, and it remains questionable whether prospective studies will ever be conducted for several reasons, mainly due to the emerging causative therapy options beyond HFT in the field. However, the use of ACEis and BBs is considered as a relative contraindication based on expert consensus opinion [24].

Due to the different underlying pathomechanisms in ATTR amyloidosis compared to common causes of HRrEF that can be treated successfully with HFT, it may seem obvious that antifibrotic treatments did not have any benefit in patients with ATTR amyloidosis, and amyloid fibril deposition appears to be the leading cause of deterioration of organ function. This might also support the use of novel treatment approaches that aim to remove amyloid from the tissue by monoclonal antibodies, namely amyloid depleters, e.g., NI006 [25], that are currently under clinical investigation.

Pathophysiologically, a potential negative effect of BBs could be explained by the negative chronotropic effect as cardiac output in restrictive hearts is maintained by heart rate [24]. ACEis might cause hypotension, especially in patients with autonomic dysfunction. Patients with ATTRwt are most commonly elderly people in the 7th decade of their life or older; thus, the presence of further (cardiac) comorbidities is very common, and therefore, it could be expected that HFT might have a positive effect in some patients with diverse causes for heart failure, which was indicated by the subgroup analysis for patients with ATTRwt and comorbidities presented in this study. Keeping in mind that no effect could be observed in ATTRv patients with comorbidities, a higher age appears to be a further factor with an impact on HFT treatment response.

This study demonstrated a shorter survival for ATTRv patients under HFT. Due to all the limitations of this retrospective study and especially the small number of ATTRv patients under HFT, the results should be interpreted with caution. However, one possible explanation might be that the above-mentioned effects of BBs and ACEis might have a negative effect on autonomic dysfunction, which is a common finding in ATTRv patients, especially in patients with neurological and mixed phenotypes. However, a subclinical neurological impairment might also be present in patients classified as having an isolated cardiac phenotype.

So far, the drug Tafamidis is the only available causative treatment option for ATTR-CM. The ATTR-ACT study demonstrated a positive effect on survival and cardiac decompensations [12], and the data were confirmed in the long-term extension study for wild-type and hereditary ATTR-CM [26]. Since the approval of Tafamidis as a first causative treatment option for patients with ATTR cardiomyopathy, either wild-type or variant, and promising opportunities for further treatment options like silencers that are currently in phase 3 studies, e.g., HELIOS-B (NCT04153149) and Cardio-TTransform (NCT04136171), as well as amyloid removal by anti-amyloid antibodies that are in early stages of clinical trials, non-specific treatment options like HFT have lost attention in the scientific field. Nevertheless, whether HFT should be initiated or not is still of major clinical importance. Based on the present data, HFT should be performed with caution in patients with ATTRv, stopped in patients with ATTRv and a mixed phenotype, and taken into consideration for ATTRwt patients with comorbidities. Future research is necessary to evaluate the potentials and risks of Angiotensin Receptor–Neprilysin Inhibitors and SGLT2 inhibitors.

### Limitations

This retrospective study has several limitations. First, the initiation of HFT was not randomized but the result of an individual decision of referring physicians. Furthermore, no data were available for patients with HFT treatment in the past that was stopped due to intolerance before their first presentation at our center. Moreover, only data from clinical routine were available, and no additional functional tests or questionnaires were available. Finally, no robust data were available for Angiotensin Receptor–Neprilysin Inhibitors or SGLT 2 inhibitors due to the low number of patients treated with these drugs.

## 5. Conclusions

The present study supports the prevailing expert opinions that HFT has no significant effect on patients with ATTR, unless other comorbidities like CAD or AHT are present. Notably, in ATTRv patients, HFT might be even harmful. The results therefore suggest a cautious use of HFT in ATTRv patients.

## Figures and Tables

**Table 1:** Baseline characteristics.

	ATTRv (n = 135)	ATTRwt (n = 268)	*p*-Value
Age (years)	63 ± 9	74 ± 6	<0.0001
Gender (n, % male)	102 (76%)	246 (92%)	<0.0001
Karnofsky Index (%)	78 ± 11	81 ± 9	0.004
aHT (n, %)	43 (32%)	167 (62%)	<0.0001
CAD (n, %)	25 (19%)	95 (35%)	0.0002
NYHA (n, %)			0.001
I:	24 (18%)	19 (7%)	
II:	54 (40%)	100 (37%)	
III:	55 (41%)	145 (54%)	
IV:	2 (1%)	4 (1%)	
TTR variants (n, %):			
p.Val40Ile	29 (21%)	-	
p.Val50Met	34 (25%)	-	
p.Val142Ile	8 (6%)	-	
p.Ile127Val	9 (7%)	-	
p.Cys30Arg	6 (4%)	-	
p.Leu78His	5 (4%)	-	
Other	44 (33%)	-	
Phenotype (n, %):			
cardiac	52 (39%)	-	
mixed (cardiac/neurologic)	83 (61%)	-	

aHT = arterial hypertension, CAD = coronary artery disease.

**Table 2:** Subgroup analysis between patients with and without HFT.

	ATTRv (n = 135)			ATTRwt (n = 268)		
	+HFT, n = 43	−HFT, n = 92	*p*-Value	+HFT, n = 140	−HFT, n = 128	*p*-Value
Age (years)	65 ± 8	62 ± 10	0.09	74 ± 7	74 ± 6	0.92
Gender (n, % male)	36 (84%)	66 (72%)	0.20	130 (93%)	116 (91%)	0.51
Karnofsky Index (%)	78 ± 11	77 ± 11	0.89	80 ± 8	82 ± 9	0.15
Systolic blood pressure (mmHg)	111 ± 22	118 ± 20	0.12	121 ± 18	125 ± 18	0.15
Diastolic blood pressure (mmHg)	72 ± 13	73 ± 13	0.57	75 ± 11	77 ± 11	0.07
LVEF (%)	40 ± 14	48 ± 16	0.01	44 ± 15	45 ± 16	0.60
IVS (mm)	19 ± 3	20 ± 4	0.12	19 ± 3	19 ± 4	0.78
NT-proBNP (ng/L)	8598 ± 17,557	3562 ± 3808	0.02	5711 ± 4943	5811 ± 9924	0.92
hsTroponin-T (pg/mL)	86 ± 143	58 ± 38	0.14	68 ± 52	60 ± 61	0.29
eGFR (mL/min × 1.73 m^2^)	69 ± 31	89 ± 33	0.002	64 ± 23	68 ± 22	0.25
Comorbidities:			0.24			0.85
aHT (n, %)	20 (47%)	23 (25%)		97 (69%)	70 (55%)	
CAD (n, %)	8 (19%)	17 (18%)		54 (39%)	41 (32%)	
NYHA (n, %)			0.12			0.42
I:	4 (9%)	20 (22%)		7 (5%)	12 (9%)	
II:	16 (37%)	38 (41%)		50 (36%)	50 (39%)	
III:	23 (53%)	32 (35%)		81 (58%)	64 (50%)	
IV:	0 (0%)	2 (2%)		2 (1%)	2 (2%)	
TTR Variants (n, %):			0.11			
p.Val40Ile	16 (37%)	13 (14%)		-	-	
p.Val50Met	8 (19%)	26 (28%)		-	-	
p.Val142Ile	3 (7%)	5 (5%)		-	-	
p.Ile127Val	3 (7%)	6 (7%)		-	-	
p.Cys30Arg	2 (5%)	4 (4%)		-	-	
p.Leu78His	1 (2%)	4 (4%)		-	-	
Other	10 (23%)	34 (37%)		-	-	
Phenotype (n, %):			0.06			
cardiac	22 (51%)	30 (33%)		-	-	
mixed (cardiac/PNP)	21 (49%)	62 (67%)		-	-	

HFT = heart failure therapy, LVEF = left ventricular ejection fraction, IVS = intraventricular septum thickness, aHT = arterial hypertension, CAD = coronary artery disease, PNP = polyneuropathy.

**Table 3:** Subgroup analysis between ATTRv patients with cardiac and mixed phenotype.

	Cardiac Phenotype			Mixed Phenotype		
	+HFT, n = 22	−HFT, n = 31	*p*-Value	+HFT, n = 21	−HFT, n = 62	*p*-Value
Age (years)	63 ± 9	62 ± 9	0.69	67 ± 6	62 ± 10	0.02
Gender (n, % male)	17 (77%)	21 (67%)	0.54	19 (90%)	45 (73%)	0.13
Karnofsky Index (%)	81 ± 7	85 ± 9	0.20	76 ± 13	75 ± 10	0.83
Systolic blood pressure (mmHg)	109 ± 21	121 ± 17	0.04	114 ± 23	117 ± 21	0.63
Diastolic blood pressure (mmHg)	70 ± 13	74 ± 12	0.21	74 ± 13	72 ± 13	0.69
LVEF (%)	40 ± 10	39 ± 15	0.86	40 ± 14	46 ± 12	0.12
IVS (mm)	18 ± 2	19 ± 3	0.29	19 ± 4	20 ± 4	0.68
NT-proBNP (ng/L)	7811 ± 8088	4208 ± 3972	0.046	9472 ± 24,418	2866 ± 3624	0.06
hsTroponin-T (pg/mL)	68 ± 39	61 ± 44	0.62	106 ± 202	51 ± 36	0.08
eGFR (ml/min)	60 ± 22	72 ± 21	0.06	79 ± 35	96 ± 35	0.06
Comorbidities:			0.45			0.46
aHT (n, %)	14 (64%)	9 (29%)		4 (19%)	14 (23%)	
CAD (n, %)	4 (18%)	6 (19%)		6 (29%)	10 (16%)	
NYHA (n, %)			0.34			0.40
I:	1 (5%)	4 (13%)		2 (10%)	11 (18%)	
II:	7 (32%)	12 (39%)		5 (24%)	19 (31%)	
III:	14 (64%)	13 (42%)		14 (67%)	31 (50%)	
IV:	0 (0%)	2 (6%)		0 (0%)	0 (0%)	
Mutations (n, %):			0.30			0.48
p.Val40Ile	13 (59%)	9 (29%)		3 (14%)	4 (6%)	
p.Val50Met	1 (5%)	3 (10%)		7 (33%)	23 (37%)	
p.Val142Ile	2 (9%)	5 (16%)		1 (5%)	0 (0%)	
p.Ile127Val	0 (0%)	0 (0%)		3 (14%)	6 (10%)	
p.Cys30Arg	1 (5%)	2 (6%)		0 (0%)	2 (3%)	
p.Leu78His	0 (0%)	0 (0%)		1 (5%)	4 (6%)	
Other	5 (23%)	12 (39%)		6 (29%)	23 (37%)	

HFT = heart failure therapy, LVEF = left ventricular ejection fraction, IVS = intraventricular septum thickness, aHT = arterial hypertension, CAD = coronary artery disease.

## Data Availability

The original contributions presented in the study are included in the article, further inquiries can be directed to the corresponding author.

## References

[1] Garcia-Pavia P., Dominguez F., Gonzalez-Lopez E. (2021). Transthyretin amyloid cardiomyopathy. Med. Clin..

[2] Jaiswal V., Agrawal V., Khulbe Y., Hanif M., Huang H., Hameed M., Shrestha A.B., Perone F., Parikh C., Gomez S.I. (2023). Cardiac amyloidosis and aortic stenosis: A state-of-the-art review. Eur. Heart J. Open.

[3] Mohammed S.F., Mirzoyev S.A., Edwards W.D., Dogan A., Grogan D.R., Dunlay S.M., Roger V.L., Gertz M.A., Dispenzieri A., Zeldenrust S.R. (2014). Left ventricular amyloid deposition in patients with heart failure and preserved ejection fraction. JACC Heart Fail..

[4] McDonagh T.A., Metra M., Adamo M., Gardner R.S., Baumbach A., Bohm M., Burri H., Butler J., Čelutkienė J., Chioncel O. (2023). 2023 Focused Update of the 2021 ESC Guidelines for the diagnosis and treatment of acute and chronic heart failure. Eur Heart J..

[5] Griffin J.M., Rosenblum H., Maurer M.S. (2021). Pathophysiology and Therapeutic Approaches to Cardiac Amyloidosis. Circ. Res..

[6] Gillmore J.D., Damy T., Fontana M., Hutchinson M., Lachmann H.J., Martinez-Naharro A., Quarta C.C., Rezk T., Whelan C.J., Gonzalez-Lopez E. (2018). A new staging system for cardiac transthyretin amyloidosis. Eur. Heart J..

[7] Grogan M., Scott C.G., Kyle R.A., Zeldenrust S.R., Gertz M.A., Lin G., Klarich K.W., Miller W.L., Maleszewski J.J., Dispenzieri A. (2016). Natural History of Wild-Type Transthyretin Cardiac Amyloidosis and Risk Stratification Using a Novel Staging System. J. Am. Coll. Cardiol..

[8] Maurer M.S., Elliott P., Comenzo R., Semigran M., Rapezzi C. (2017). Addressing Common Questions Encountered in the Diagnosis and Management of Cardiac Amyloidosis. Circulation..

[9] Coelho T., Maia L.F., Martins da Silva A., Waddington Cruz M., Plante-Bordeneuve V., Lozeron P., Suhr O.B., Campistol J.M., Conceição I.M., Schmidt H.H.-J. (2012). Tafamidis for transthyretin familial amyloid polyneuropathy: A randomized, controlled trial. Neurology.

[10] Adams D., Gonzalez-Duarte A., O’Riordan W.D., Yang C.C., Ueda M., Kristen A.V., Tournev I., Schmidt H.H., Coelho T., Berk J.L. (2018). Patisiran, an RNAi Therapeutic, for Hereditary Transthyretin Amyloidosis. N. Engl. J. Med..

[11] Benson M.D., Waddington-Cruz M., Berk J.L., Polydefkis M., Dyck P.J., Wang A.K., Planté-Bordeneuve V., Barroso F.A., Merlini G., Obici L. (2018). Inotersen Treatment for Patients with Hereditary Transthyretin Amyloidosis. N. Engl. J. Med..

[12] Maurer M.S., Sultan M.B., Rapezzi C. (2019). Tafamidis for Transthyretin Amyloid Cardiomyopathy. N. Engl. J. Med..

[13] McDonagh T.A., Metra M., Adamo M., Gardner R.S., Baumbach A., Böhm M., Burri H., Butler J., Čelutkienė J., Chioncel O. (2022). 2021 ESC Guidelines for the diagnosis treatment of acute chronic heart failure: Developed by the Task Force for the diagnosis treatment of acute chronic heart failure of the European Society of Cardiology (ESC) With the special contribution of the Heart Failure Association (HFA) of the ESC. Eur. J. Heart Fail..

[14] Heidenreich P.A., Bozkurt B., Aguilar D., Allen L.A., Byun J.J., Colvin M.M., Deswal A., Drazner M.H., Dunlay S.M., Evers L.R. (2022). 2022 AHA/ACC/HFSA Guideline for the Management of Heart Failure: A Report of the American College of Cardiology/American Heart Association Joint Committee on Clinical Practice Guidelines. Circulation.

[15] Pitt B., Pfeffer M.A., Assmann S.F., Boineau R., Anand I.S., Claggett B., Clausell N., Desai A.S., Diaz R., Fleg J.L. (2014). Spironolactone for heart failure with preserved ejection fraction. N. Engl. J. Med..

[16] Anker S.D., Butler J., Filippatos G., Ferreira J.P., Bocchi E., Bohm M., Brunner–La Rocca H.-P., Choi D.-J., Chopra V., Chuquiure-Valenzuela E. (2021). Empagliflozin in Heart Failure with a Preserved Ejection Fraction. N. Engl. J. Med..

[17] Ioannou A., Massa P., Patel R.K., Razvi Y., Porcari A., Rauf M.U., Jiang A., Cabras G., Filisetti S., E Bolhuis R. (2023). Conventional heart failure therapy in cardiac ATTR amyloidosis. Eur. Heart J..

[18] Garg R., Yusuf S., Bussmann W.D., Sleight P., Uprichard A., Massie B., McGrath B., Nilsson B., Pitt B., Magnani B. (1995). Overview of randomized trials of angiotensin-converting enzyme inhibitors on mortality and morbidity in patients with heart failure. J. Am. Med. Assoc..

[19] Braunwald E. (2022). SGLT2 inhibitors: The statins of the 21st century. Eur. Heart J..

[20] Heidenreich P.A., Bozkurt B., Aguilar D., Allen L.A., Byun J.J., Colvin M.M., Deswal A., Drazner M.H., Dunlay S.M., Evers L.R. (2022). 2022 AHA/ACC/HFSA Guideline for the Management of Heart Failure: Executive Summary: A Report of the American College of Cardiology/American Heart Association Joint Committee on Clinical Practice Guidelines. J. Am. Coll. Cardiol..

[21] Patoulias D., Papadopoulos C., Doumas M. (2022). “SGLT2i in patients with transthyretin cardiac amyloidosis, a well-tolerated option for heart failure treatment? Results from a small, real-world, patients series” comment. Intern. Emerg. Med..

[22] Dobner S., Bernhard B., Asatryan B., Windecker S., Stortecky S., Pilgrim T., Gräni C., Hunziker L. (2023). SGLT2 inhibitor therapy for transthyretin amyloid cardiomyopathy: Early tolerance and clinical response to dapagliflozin. ESC Heart Fail..

[23] Gonzalez-Lopez E., Gallego-Delgado M., Guzzo-Merello G., de Haro-Del Moral F.J., Cobo-Marcos M., Robles C., Bornstein B., Salas C., Lara-Pezzi E., Alonso-Pulpon L. (2015). Wild-type transthyretin amyloidosis as a cause of heart failure with preserved ejection fraction. Eur. Heart J..

[24] Rubin J., Maurer M.S. (2020). Cardiac Amyloidosis: Overlooked, Underappreciated, and Treatable. Annu. Rev. Med..

[25] Garcia-Pavia P., Siepen F.A.D., Donal E., Lairez O., van der Meer P., Kristen A.V., Mercuri M.F., Michalon A., Frost R.J., Grimm J. (2023). Phase 1 Trial of Antibody NI006 for Depletion of Cardiac Transthyretin Amyloid. N. Engl. J. Med..

[26] Rapezzi C., Elliott P., Damy T., Nativi-Nicolau J., Berk J.L., Velazquez E.J., Boman K., Gundapaneni B., Patterson T.A., Schwartz J.H. (2021). Efficacy of Tafamidis in Patients with Hereditary and Wild-Type Transthyretin Amyloid Cardiomyopathy: Further Analyses From ATTR-ACT. JACC Heart Fail..

